# Aneurism of Aorta, with Notes of a Clinical Lecture, by Prof. H. A. Johnson, Attending Physician

**Published:** 1869-12

**Authors:** Wm. E. Quine

**Affiliations:** House Physician


### COOK COUNTY HOSPITAL.

### ANEURISM OF AORTA,

WITH NOTES OF A CLINICAL LECTURE, BY PROF. H. A. JOHNSON, ATTENDING PHYSICIAN.

By WM. E. QUINE, M.D., House Physician.

*History.*—Jeremiah C., *æt.* 46, Ireland, blacksmith; admitted Sept. 30th, 1869. Has been more or less subject to rheumatism during the last twelve years. One year ago, complained of pain in both mammary regions, which (four months later) was accompanied by dyephagia, dyepnœa, sore throat, hoarseness, incessant cough, and copious frothy expectoration. To these symptoms, which are rapidly increasing in severity, there is now added anorevia, great feebleness, and profuse diarrhœa.

*Examination—Inspection.*—Moderate emaciation, lividity of skin, features expressive of great anxiety, remarkable prominence of third, fourth, and fifth right costal cartilages, elevation of left shoulder, and diminished respiratory movements of left side.

*Mensuration.*—Circumferential measurements of left side, a half inch greater than of corresponding regions of right.

*Palpation.*—Vocal fremitus exaggerated on right side, absent on left. Left intercostal depression on inspiration. Prolonged heaving impulse against third, fourth, and fifth right costal cartilages, coincident with ventricular systole. Apex of heart beats two inches below, and one inch outside left nipple.

*Percussion.*—Volume of resonance of left side increased. Percussion note of right infra clavicular region is of higher pitch and less intensity than natural; over third, fourth, and fifth, right costal cartilages flat; and over remainder of right side, natural.

*Auscultation.*—In right infra clavicular region the inspiratory murmur is irregular and jerking, and the epiratory prolonged; in remainder of right chest, puerile respiration. Over left side, respiratory murmur is absent, except during forced inspiratory efforts. Vocal resonance diminished on left side; increased on right. Friction sounds, coincident with both sounds of heart, are heard at its apex, and above notch of sternum. A low, musical, prolonged murmur, heard best over second, third, and fourth right costal cartilages, and not at all in back, follows immediately the second sound of heart.

NOTES OF CLINICAL LECTURE BY PROF. JOHNSON, OCT. 5TH, 1869.

*Diagnosis.*—The history and physical signs point to a lesion of thoracic viscera, which interferes with performance of their functions. What is its nature? Is absence of respiratory murmur in left lung due to tuberculous or cancerous infiltration, or to fluid in pleural cavity? No. Because this lung is even more resonant than the other. Is it due to occlusion of trachea, or of left bronchus? Cannot be trachea, because right lung performs its function. Must be bronchus. Is this occlusion caused by foreign body within bronchus, or by pressure upon it. Must be pressure upon it; because by forced efforts left lung can be inflated, and such efforts would cause foreign body to become more tightly wedged, and the more perfectly prevent entrance of air. Is the prominence of right costal cartilages, due to displacement of heart by emphysema of left lung, or by fluid in left pleural cavity, or by ascites, or an abdominal tumor? No. Because apex of heart is further to left of sternum, and lower than natural. Is the heaving impulse against right costal cartilages due to hydrops pericardii, or hypertrophy of heart, with dilitation? No. Because, as in first instance, there is no diffuse impulse, and no triangular area of dulness; because cardiac sounds are much louder than natural; and because of heaving impulse to right of sternum. A heart does not generally dilate in this direction, and never attains this magnitude. Therefore, because of the small thready pulse, prominence of right cartilages, pressure upon left bronchus, and œsophagus (all of which are in relation with aorta), and prolonged heaving impulse against right cartilages, with murmur, we are justified in calling this aneurism of aorta.

*Causes.*—Aneurism never occurs in this region, except as the result of previous disease of arterial coats. Whether the disease consists in the distinct deposit of patches of atheroma, of fat globules in the interstices, or of replacement of fat for normal elements, the natural elasticity becomes lost, proportionately to amount of change. Hence, the artery becomes less able to contract on its contents, and yields to the repetition of shocks which it sustains by ventricular contractions. Rheumatism, and gout, very frequently precede aneurism, and it is a question in this case whether the dilitation is due to a deposit, similar to that which has probably taken place in apex of right lung, and there called tubercle, or to a deposit similar to that which occurs in and around his joints. Age exercises a powerful predisposing influence, as it is rarely met with in childhood and advanced life.

*Kinds.*—The kinds which usually affect the aorta, are the fusiform, and false sacculated. The first consists not only in equal expansion of all the coats through the whole circumference of the vessel, but there is positive elongation as well. The second consists in a tumor that springs from side of vessel, with interior of which it communicates by a small aperture, and is caused by rupture of one or two of its coats with dilitation of others. Not unfrequently the latter springs from side of former. Judging from prominence of right cartilages, from evident pressure on left bronchus, and œsophagus, from absence of loud rasping murmur, and well-marked vibration, we have in this case a fusiform aneurism involving the whole arch.

*Prognosis*—Is of course grave. Although spontaneous cures sometimes occur in vessels of smaller size, by the gradual deposition of laminated fibrin within sac, which may result in occlusion, it is not to be expected in one of this magnitude, but rather that the patient will finally succumb to exhaustion caused by pressure upon, and consequent interference with, the functions of important parts, or to hæmorrhage, caused by rupture of sac externally, or into trachea, or bronchi, or œsophagus, or pleural, or pericardial cavity.

*Treatment.*—The most we can hope to accomplish is the palliation of the more distressing symptoms, of the disease and retardation of its progress; and this can be best effected by arterial sedatives to lessen the force of the heart's impulse, so as to diminish pressure upon arterial coats, and nerve sedatives, to diminish sensibility and procure rest. In this case, profuse diarrhœa (probably the peripheral expression of central obstruction to circulation) is an urgent symptom, and also the cough, and dyspnœa.

℞. Acidi Tanici,---------------------------------gr. iij.
Tinct. Opii,-------------------gtt. x tertia-hora, and

℞. Tinct. Verat. Virid. }

Acid Hydrodcyanici, } āā---------------gtt. iv.

also, every three hours.

*Autopsy.*—On reflecting the soft parts, there was evident prominence of third, fourth, and fifth right costal cartilages, with no loss of tissue. Tubercle in apex of right lung. Visceral and parietal layers of pericardium firmly adherent. A fusiform aneurism arose from that portion of aorta contained within pericardium, and extended to eighth dorsal vertebra. Tough decolorized fibrin adhered to some parts of the walls, and to others deposits of apparently more recent date, as indicated by the retention of red coloring matter. A large stellate opening from sac into the œsophagus. Stomach distended with dark fluid blood, and friable clots. Part of bodies of sixth, seventh, and eighth dorsal vertebræ absorbed. The heart was hypertrophied, but could not be weighed, as it was kept attached to specimen.

